# Coarse-graining protein structures into their dynamic communities with DCI, a dynamic community identifier

**DOI:** 10.1093/bioinformatics/btac159

**Published:** 2022-03-17

**Authors:** Ambuj Kumar, Pranav M Khade, Karin S Dorman, Robert L Jernigan

**Affiliations:** Bioinformatics and Computational Biology Program, Iowa State University, Ames, IA 50011, USA; Roy J. Carver Department of Biochemistry, Biophysics and Molecular Biology, Iowa State University, Ames, IA 50011, USA; Bioinformatics and Computational Biology Program, Iowa State University, Ames, IA 50011, USA; Roy J. Carver Department of Biochemistry, Biophysics and Molecular Biology, Iowa State University, Ames, IA 50011, USA; Bioinformatics and Computational Biology Program, Iowa State University, Ames, IA 50011, USA; Department of Statistics, Iowa State University, Ames, IA 50011, USA; Bioinformatics and Computational Biology Program, Iowa State University, Ames, IA 50011, USA; Roy J. Carver Department of Biochemistry, Biophysics and Molecular Biology, Iowa State University, Ames, IA 50011, USA

## Abstract

**Summary:**

A new dynamic community identifier (DCI) is presented that relies upon protein residue dynamic cross-correlations generated by Gaussian elastic network models to identify those residue clusters exhibiting motions within a protein. A number of examples of communities are shown for diverse proteins, including GPCRs. It is a tool that can immediately simplify and clarify the most essential functional moving parts of any given protein. Proteins usually can be subdivided into groups of residues that move as communities. These are usually densely packed local sub-structures, but in some cases can be physically distant residues identified to be within the same community. The set of these communities for each protein are the moving parts. The ways in which these are organized overall can aid in understanding many aspects of functional dynamics and allostery. DCI enables a more direct understanding of functions including enzyme activity, action across membranes and changes in the community structure from mutations or ligand binding. The DCI server is freely available on a web site (https://dci.bb.iastate.edu/).

**Supplementary information:**

Supplementary data are available at *Bioinformatics* online.

## Graphical Abstract

**Figure:**
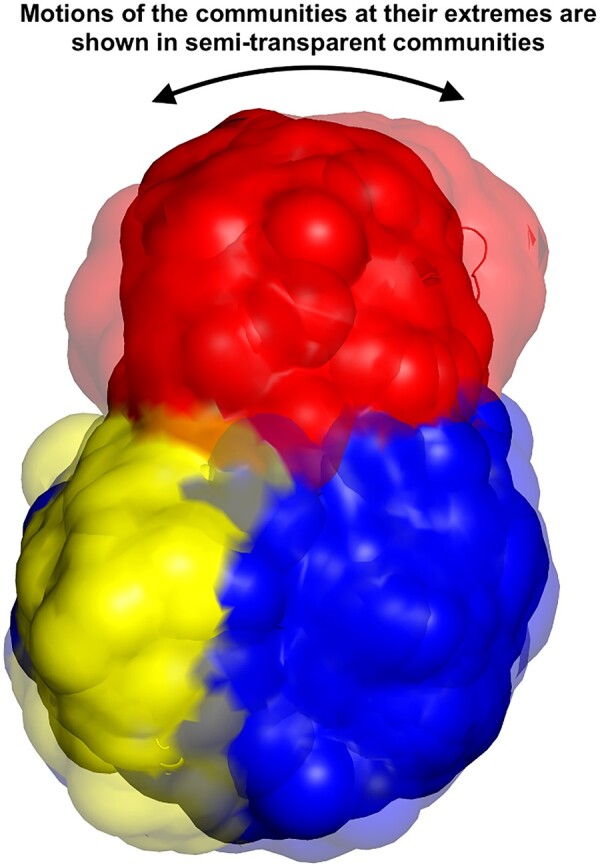
Three communities for Zika virus NS5 protein (PDB ID: 5M2Z). Each color represents a unique community with its motions.

## 1 Introduction

Since the first protein structure of myoglobin was determined, there has been a struggle to interpret protein structures in terms of their functions (Fersht, 2008; Kendrew *et al.*, 1958), even though there has long been a widespread consensus that dynamics is key to such an understanding. But a simple interpretation of dynamics from structure has not been available, and protein researchers have been saddled with interpreting the complexities and randomness manifested in atomic molecular dynamics simulations. Recently, there has been a simpler comprehension of the range of motions available to any given protein structure, constrained simply by the geometry of a particular structure, by using elastic network models (Atilgan *et al.*, 2001; Bahar *et al.*, 1997; Tirion, 1996). Recent progress is providing simple ways to comprehend protein dynamics based on computing the cohesiveness of different parts of a protein structure, which is based on the local packing densities (Khade *et al.*, 2019, Khade, 2021). And this has the important advantage of leading to simple ways of visualizing the protein dynamics.

The approach taken is to identify the most rigid parts of a structure and how they move, i.e. the groups of amino acids that move in the most coherent ways, which are naturally the most rigid parts of a structure. This coarse-graining approach for interpreting protein structures significantly simplifies the understanding of dynamics, making the most important functional motions significantly clearer, and usually leads to a view of dynamics in terms of the most essential dynamics required for function. This is clearly an approximation that overlooks some local details of dynamics but is justified by the simpler comprehension of protein function provided. Usually this provides an essential view of how dynamics relates to function. The functional motions are then the changes in the relative positions and orientations among these communities with respect to one another. In this way the identification of the most coherent groups of amino acids enables the identification of the most important characteristic motions of any given protein. This is an approach that was first demonstrated by using the Gaussian elastic network model (GNM) (Bahar *et al.*, 1997) by Yesylevskyy *et al.* (2006) to identify domains and then further articulated by McClendon *et al.* (2014), in their kinase studies where they identified these communities based on molecular dynamics simulations. More recently our own studies of these communities (Mishra and Jernigan, 2018) has further validated the use of dynamics from the coarse-grained GNM. While the coarse graining with these elastic network models is usually taken as a uniform coarse-graining with a single geometric point for each amino acid, the second level of coarse-graining described in these dynamics communities coarse-grains further, based primarily on the packing densities within structures. The motion correlations among all residues are well captured by identifying those residues moving collectively within the dynamic communities. Applications of this approach have included allosteric regulation (Kornev and Taylor, 2015; Yao *et al.*, 2016), detection of mutationally induced changes in protein structure and dynamics (Chopra *et al.*, 2016; Mishra and Jernigan, 2018), signal transmission (Chopra *et al.*, 2016), identification of cancer mutational hotspots (Kumar *et al.*, 2019), enzyme regulatory mechanisms (McClendon *et al.*, 2014) and understanding of how mutants can interfere with dynamics by significant changes in the way in which the structure is distributed into these communities (Chopra *et al.*, 2016; McClendon *et al.*, 2014).

Elastic network model (ENM) have been widely implemented to study the characteristic motions of a protein (Yang *et al.*, 2007, 2009). Correlations among residues can be obtained from ENMs and have been widely used to derive the functional motions of proteins. We have previously used these cross-correlation matrices from the Gaussian Network Model (GNM) to estimate the protein dynamics communities (Mishra and Jernigan, 2018) using manual pruning of hierarchical trees. Moreover, implementation of GNM and hierarchical clustering for detecting protein dynamic communities have also been implemented in Hierarchical Clustering of the Correlation Patterns (HCCP) (Yesylevskyy *et al.*, 2006). Here, we develop a new automated protein dynamics community identifier based on GNM, Euclidian dynamic distance measure and hierarchical clustering. Since dynamics communities, are dependent on residue dynamical cross-correlations, we can map out the dynamic allostery across the whole structure. Data-driven predictions of the optimal number of communities enable DCI to predict communities corresponding to known functional domain in proteins (see Results and Discussion). Such predictions are challenging through HCCP and Mishra and Jernigan (2018), where the optimal number of communities are unknown, and therefore it does not always lead to the correct boundaries between communities.

The aim of this article is to further demonstrate the utility of this approach and its ability to explain many important functional aspects of dynamics, including but not limited to, both for globular proteins, as well as for membrane proteins, where the communities are found to be quite extended in shape and spanning across the membrane. Another intention of the present work is to make the approach more accessible to a broad group of users across the many different categories of protein researchers.

## 2 Materials and methods

### 2.1 Data collection

Protein structures were collected from the Protein Databank (PDB) (Berman, 2000). Protein domain annotations were collected from SCOP database (Andreeva *et al.*, 2014, 2020). Cryptic pocket data was collected from Cimermancic et al. (2016).

### 2.2 Dynamic distance matrix calculation

Elastic network models capture the characteristic dynamics of a protein. GNM, in particular, was constructed to study the scalar fluctuations of a molecular structures and it has been used to study motions (Rader *et al.*, 2005; Yang *et al.*, 2009) as well as allostery (Kaynak *et al.*, 2020). In coarse-grained GNM, harmonic potentials between any two close residues (i and j**)** within a C^α^ cutoff distance of 7.0 Å, connected with a spring with force constant γ, is calculated a*s*
 

(1) V=12γ∑i, jNΓijΔRi-ΔRj2 ,

where *N* is the total number of residues, γ=1.0, $\Delta R_{i}$ and $\Delta R_{j}$ are displacements of residues i and j from their equilibrium positions, and Γ is the N×N connectivity matrix

(2) Γij=-1 ####if i≠j and Rij≤7.0Å0#if i≠j and Rij>7.0Å-∑i, i ≠jΓij#if i=j  ,

here, $R_{ij}$ is the equilibrium distance between residues i and j. Eigenvalues (λ) and eigenvectors (u) obtained by singular value decomposition of Γ are used to calculate the pseudoinverse $\Gamma^{-1}$ as

(3) Γ-1=∑i=2N1λiuiuiT ,

here, the first eigenvector and eigenvalue are not included in the calculation since they correspond to the rigid body motion of the protein. The cross-correlation values $C_{ij}$ are calculated a*s*
 

(4) Cij=Γij-1Γii-1×Γjj-1 

these form the matrix C for all pair correlations. The Euclidian dynamic distance between residue i and j ($D_{ij}$) for a protein is defined by

(5) Dij=21-Cij ,

where $D_{ij}$ forms dynamic distance matrix D for all residue pairs.

### 2.3 Dynamic community detection

Agglomerative hierarchical clustering (https://scikit-learn.org/stable/modules/generated/sklearn.cluster.AgglomerativeClustering.html) (Blondel *et al.*, 2011; Contreras and Murtagh, 2015) using Ward linkage was implemented on the dynamic distance matrix D to generate residue clusters forming dynamic communities. Here, all observations are first represented as a hierarchical tree, which represents their relationship. In agglomerative hierarchical clustering, initially each observation starts with its own cluster and pairs of clusters that are then merged iteratively, as we move upward in the hierarchy. The desired number of clusters can be obtained by pruning the tree using a cutoff. Here, we prune the tree iteratively to obtain 2–20 number of communities. The maximum iteration value is set to 20 as a default parameter, which can be increased by the user to any number up to the maximum number of residues in the protein. Greater number of community iterations allows DCI to explore larger number of communities with relatively smaller residue clusters. The optimal number of clusters was determined by calculating the Calinski and Harabasz (CH) score (Calinski and Harabasz, 1974) applied to the clusters generated in the *n*th (connect it with the cluster number) iteration of hierarchical clustering and the matrix D. The Calinski-Harabasz score calculates the ratio of the variance of the sums of squares of the distances of individual objects to their cluster center as the sum of squares of the distances between the cluster centers. The community iteration with the highest CH score is chosen as the optimal dynamic community distribution for any given protein.

### 2.4 Protein motion generation

The anisotropic network model (Atilgan *et al.*, 2001) is another elastic network model, primarily used to study the protein motion directions. Here, the C^**α**^ atoms of each residue within a distance cutoff of 15 Å are connected with springs. The Hessian matrix is a 3N×3N matrix containing second derivatives of the potential with respect to position. Singular value decomposition of the Hessian matrix yields 3N-6 eigenvectors ν representing internal motions and excludes six rigid body motions with zero eigenvalues. For the *i*^th^ eigenvector, the corresponding motion of protein is calculated as,

(6) R'=R+s νi ,

where $R^{'}$ is the *x*, *y* and *z* coordinate of the protein residues displaced along the direction of the *i*th eigenvector, R is the *x*, *y* and *z* coordinate of the protein residues at initial state and s is the amplification parameter, starting from the initial state moving progressively further along the *i*th eigenvector.

## 3 Results and discussion

Earlier, the application of the Girvan–Newman (Newman and Girvan, 2002) network clustering algorithm on the dynamic graph models constructed using Molecular Dynamics (MD) simulations has been widely implemented to obtain the protein dynamic communities (Ahuja *et al.*, 2019; Atilgan *et al.*, 2021; McClendon *et al.*, 2014). The Girvan–Newman algorithm generates communities in a network by removing edges that lie between the highly connected regions. Since the application of the Girvan–Newman algorithm to MD-generated ensembles of structures is largely dependent on a network generated using residue physical contacts, it will often underestimate the contribution of physically distant residue cross-correlations. Here, we use dynamic cross-correlation between residues to generate communities where even physically distant residues are grouped together when they exhibit strong motion correlations. Our community modeling approach detects those physically distant residues involved in dynamic allostery.

**Fig. 1.**
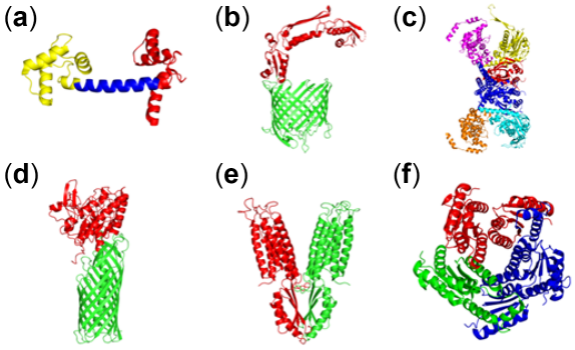
Protein dynamic communities identified with the present DCI approach. (**a**) Three communities in calmodulin (PDB ID: 1EXR, Supplementary Fig. S1), (**b**) two communities in the translocation and assembly module TAMA protein (PDB ID: 4C00, Supplementary Fig. S2), (**c**) six communities in glycyl-tRNA synthetase (PDB ID: 7EIV, Supplementary Fig. S3), (**d**) two communities in the autotransporter EstA (PDB ID: 3KVN, Supplementary Fig. S4), (**e**) two zinc transporter YiiP (PDB ID: 3H90, Supplementary Fig. S5) and (**f**) three communities in 6,7-dimethyl-8-ribityllumazine synthase (PDB ID: 2A58, Supplementary Fig. S6). Here, each color represents a different community

DCI is a protein residue community detection algorithm, which can either estimate the optimal number of communities directly from the data, or the optimal number of communities can also be specified by the user. It is designed to capture the dynamic communities within a protein structure, to detect closely packed residues including those distantly located residues. Here, we present a selection of dynamic communities for six proteins (Fig. 1). Results from calmodulin show three distinct communities, which represent the N-terminal and C-terminal domains separated by central hinge region (shown in blue). The Supplementary Movies S1 and S2 show who it acts as a flexible linker and assists in the domain rotations as well as the open-closed conformational transition. Similarly, the N-terminus of translocation and assembly module A (TAMA) protein and the passenger domain of autotransporter Esterase (EstA) have unique motions.

### 3.1 Protein domain prediction using DCI

Protein domains are the fundamental functional units of proteins. Our result indicate that DCI can identify the known functional domains within protein. Out of 98 globular protein domains obtained from the SCOP database, DCI was able to generate a separate community for 73 domains (Supplementary Table S1), by using the optimal number of community parameter estimated using the CH Score. Moreover, DCI generated separate communities for 23 out of the 25 remaining domains when the number of communities is larger than optimal number of communities (Supplementary Table S1). The trend of being able to capture functional domains in a protein as a community indicates the ability of DCI to represent the important structure-function relationships in proteins.

### 3.2 Cryptic pocket comprises multiple communities

**Fig. 2.**
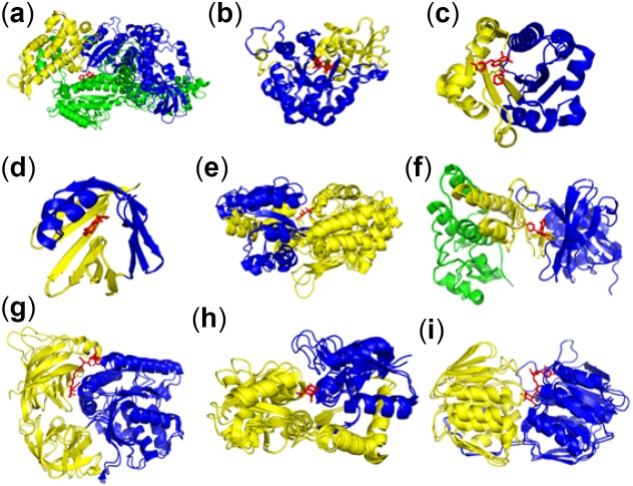
Cryptic pockets in proteins where the binding ligands are shown in red. Here, for each protein, both apo and holo states are superimposed on top of on one another, showing the dynamic communities, predicted using the apo state structure, with communities shown in blue, yellow and green: (**a**) myosin II heavy chain (PDB ID: 2AKA), (**b**) Chitinase (PDB ID: 3CHE), (**c**) integrin alpha-L (PDB ID: 3F74), (**d**) adipocyte lipid-binding protein (PDB ID: 1ALB), (**e**) Maltose-binding periplasmic protein (PDB ID: 3PUW), (**f**) hepatocyte growth factor receptor (PDB ID: 1R1W), (**g**) elongation factor U (PDB ID: 1EXM), (**h**) glutamate receptor 2, (**i**) UDP-N-acetylglucosamine 1-carboxyvinyltransferase. A color version of this figure appears in the online version of this article

Proteins often have various ligand binding pockets that are not always accessible to the ligand free structure in the apo state conformation and may require conformational changes to allow entry of the ligand (Cimermancic *et al.*, 2016). Here, we show that such cryptic pockets usually consist of multiple communities, predicted here by DCI, in such an arrangement that allows its opening and closing to be directly connected with the motions of these sets of local communities (Fig. 2, Supplementary Table S2). The relationship between opening and closing of cryptic pockets and the corresponding DCI community arrangement occurs due to the correlated motions of individual cryptic pocket residues, which are motions within the pocket among the specific community junctions. Therefore, identifying the DCI community arrangements can help to predict whether a pocket, although not visible in the apo state, can undergo a conformational transition to open and enable the entry of ligand.

### 3.3 Community boundaries indicate the hinge location for open-closed transitions

**Fig. 3.**
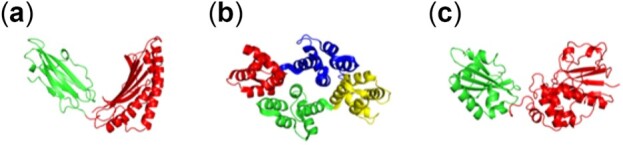
Communities neighboring central hinges regions where the hinge bending leads to conformational transitions essential to function. (**a**) Two communities in HLA class I histocompatibility antigen (PDB ID: 1ZSD, Supplementary Fig. S7), (**b**) four communities in Annexin protein (PDB ID: 1MCX, Supplementary Fig. S8) and (**c**) two communities in Inorganic pyrophosphatase (PDB ID: 1K23, Supplementary Fig. S9). Here, each color represents a different community. Open–close transitions are observed across the boundary of communities in each protein

Results from HLA class I histocompatibility antigen, annexin and Inorganic pyrophosphatase (Fig. 3) demonstrate protein community identities that split the protein across the axis of open and closed conformational transitions (Supplementary Movies S3–S5). Such an arrangement of communities around the center of a flexible linker where the bending/rotation causes the open-closed transition in a protein helps to find the residues where the protein global motions occur. Protein open-closed movies were generated to observe the specific relationships of the communities to functional motions of the proteins. A clear representation of opening and closing of the structure along the community boundaries is clearly seen for each protein (Supplementary Movies S3–S5), indicating that the axis of hinge bending resides along the open-closed motion boundary and can be predicted with our dynamic community prediction algorithm. Annexin monomer structure forms a twofold symmetric arrangement of the four similar domains separated by a groove (Cregut *et al.*, 1998). Each of the annexin domains was identified as a distinct community by DCI (Fig. 3b). Opening and closing of annexin is mediated by bending of the domain across its central groove (Cregut *et al.*, 1998) as shown in the movie (Supplementary Movie S4), indicating that DCI can capture the community of residues which move in a correlated motion within a biological mechanism.

### 3.4 Hemoglobin

**Fig. 4.**
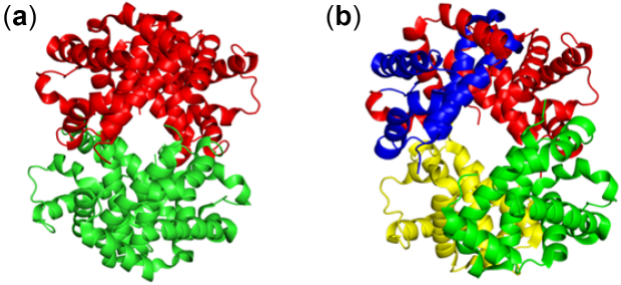
Conformational transition within dynamic communities of hemoglobin with the corresponding changes in community structure. (**a**) Two communities in deoxy hemoglobin (PDB ID: 1HV4, Supplementary Fig. S10) and (**b**) four communities in oxy hemoglobin (1GZX, Supplementary Fig. S11). Here, each color represents a unique community. Here, the communities are calculated from their corresponding crystal structures after removing all bound oxygens and hemes. Root mean-square-distance between the two ligand free crystal structures is 2.68 Å

Oxygen binds to hemoglobin in a cooperative process, where the binding of oxygen to one subunit leads to an increase in oxygen binding affinity of other subunits, shifting the hemoglobin conformation from the R-state with no oxygen bound to the T-state with oxygen bound to all four subunits. Here, we have used the oxygen-free crystal structure of the R and the T states to calculate the coarse-grained dynamic communities from their corresponding crystal structures. Our results (Fig. 4) show that these two cases are significantly different, with two communities in the R-state where each community contains one alpha and one beta monomer; whereas in the T-state it contains four communities, indicating that binding of oxygen to all four chains leads to increased degrees of freedom in the hemoglobin tetramer.

### 3.5 G-Protein coupled receptors

**Fig. 5.**
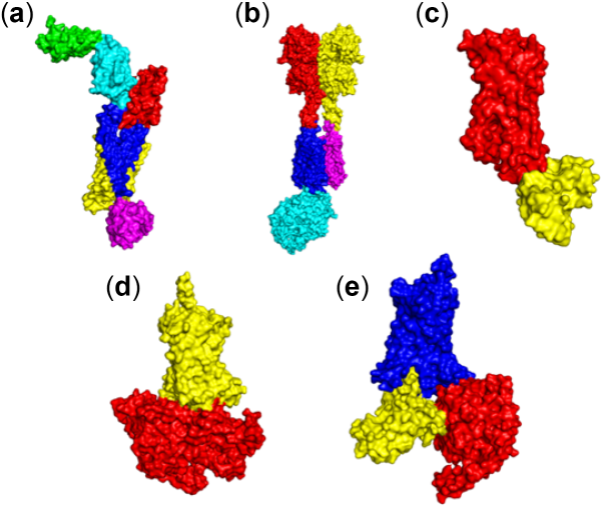
Dynamic communities of some membrane proteins: (**a**) six communities in the Glucagon class B G protein-coupled receptor (PDB ID: 5XF1, Supplementary Fig. S12), (**b**) five communities in the Metabotropic glutamate receptor 2 in complex with guanine nucleotide-binding protein complex (PDB ID: 7MTS, Supplementary Fig. S13), (**c**) two communities in the lipid G protein-coupled receptor (PDB ID: 3D4S, Supplementary Fig. S14), (**d**) two communities in the corticotropin-releasing factor receptor 1 protein complex (PDB ID: 6P9S, Supplementary Fig. S15), (**e**) three communities in the human cholecystokinin 1 receptor (PDB ID: 7MBY, Supplementary Fig. S16). Here, each color represents a unique community. Here, membrane and ligand atoms are not included in the dynamic community calculations

G-protein coupled receptors (GPCR) are the most studied and most diverse group of membrane bound proteins, which are an essential component of many cell signaling cascades. It regulates diverse signaling cascades across the membrane (Latorraca *et al.*, 2017). Transfer of signal across the membrane requires effective conformational changes. Different segments of the protein undergo unique rearrangements, leading to a large-scale conformational change associated with the signal transduction. Study of dynamic communities in GPCRs can help us to identify the domains in the protein that undergo motions, enabling the entry of ligand or activation of GTP binding and signal transmittal. Our coarse-grained elastic network model captures distinct and uniquely packed domains, such as transmembrane regions and different extracellular and intracellular domains as separate communities (Fig. 5), which may undergo unique motions to initiate the characteristic conformational transitions associated with signal transduction.

**Fig. 6.**
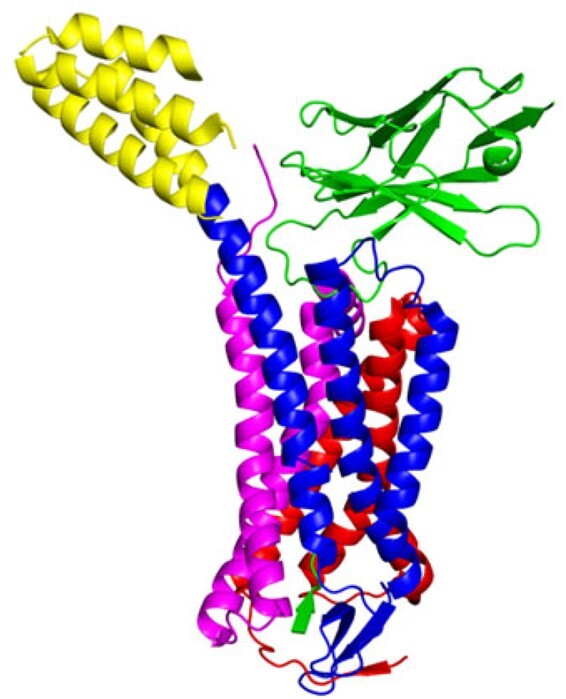
Communities in angiotensin II type 1 receptor bonded with TRV026 peptide and nanobody Nb.AT110i1_le (PDB ID: 6OS2, Supplementary Fig. S17). Here, each color represents a unique community. Nb.AT110i1_le (top green) and TRV026 peptide (bottom green) are in same community, but they are not physically connected, yet have a strong allosteric relationship. Here, AT1R has communities colored in blue, red, magenta and yellow. A color version of this figure appears in the online version of this article

GPCR proteins are one of the most frequently studied cases of allosteric signal transduction for drug design. Here we generated community arrangements of angiotensin II type 1 receptor GPCR protein bonded to the allosteric effectors TRV026 peptide and nanobody Nb.AT110i1_le. It has been shown that the synthetic nanobody Nb.AT110i1_le stabilizes the active state of AT1R (Wingler *et al.*, 2020), and increases the binding of TRV026 peptide through an allosteric relationship as reported by radioligand binding (Wingler *et al.*, 2020). Our results indicate a strong direct allosteric relationship between the TRV026 (green peptide at the bottom in Fig. 6) and Nb.AT110i1_le (green at the top right in Fig. 6) by forming a single common community including both. Formation of one common community indicates that change in the dynamics of Nb.AT110i1_le significantly affects the change in the dynamics of the TRV026 peptide, and therefore can affect its binding affinity to the receptor. Our results show that DCI can also predict the presence of allosteric relationships between distant parts in the GPCR protein complexes.

### 3.6 Allosteric regulation among membrane protein communities

**Fig. 7.**
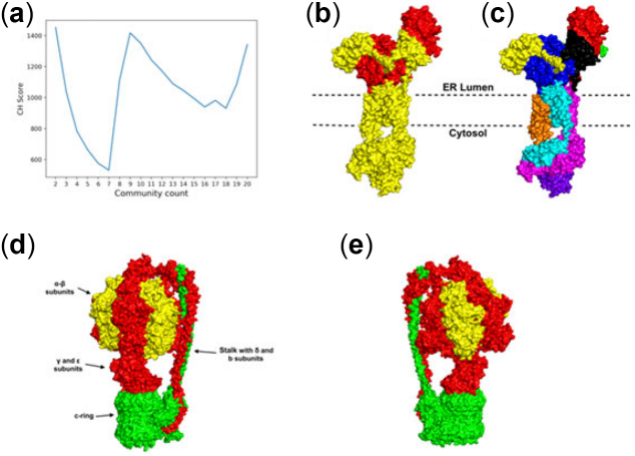
Two transmembrane assemblages (**a**) CH scores for each community in the ER membrane protein, (**b**) and (**c**) show two alternative sets of communities indicated by part (a) for the ER membrane protein (PDB ID: 6WW7), (b) two dynamic communities in the ER membrane protein complex, (c) nine dynamic communities in the ER membrane protein complex, (**d**) three dynamic communities in the ATP synthase (PDB ID: 6WNR, Supplementary Fig. S18), (**e**) backside view of the three dynamic communities in ATP synthase. Here, each color represents a unique community

Dynamic communities can be used to study the allosteric property in a protein (Ahuja *et al.*, 2019; Atilgan *et al.*, 2021; Guo and Zhou, 2015; Yao *et al.*, 2016) as shown above in AT1R. Next, we investigate allosteric communication in two membrane bound protein assemblages. The endoplasmic reticulum (ER) membrane protein complex (Fig. 7a–c) plays an important role in folding and insertion of transmembrane protein domains into the membrane (Chitwood *et al.*, 2018). Our results indicate the presence of 2 dynamic communities within the ER complex with highest CH score of 1452 (Fig. 7a), where one dynamic community (shown in yellow in Fig. 7b) is shared among the ER lumen as well as the cytosol domain. Sharing of a community by residues in cytosol, transmembrane domain and ER lumen indicates a strong dynamical cross-correlation, and therefore strong allosteric communication among the domains across the membrane. Prevalence of this allosteric relationship within the protein was further supported by the second-best CH score (1418) forming 9 communities (Fig. 7a and c). Our results show that the allosteric relationship between the transmembrane and cytosol domains persists even when the protein is alternatively divided into 9 smaller communities (Fig. 7b). Such allosteric communication may play a key role in regulating the ER membrane protein complex function.

A similar trend of dynamic allostery is observed in ATP synthase (Fig. 7d and e), where residues of same community are found in the γ and ε subunit along with the physically distant b subunit of the stalk domain as well as α subunits of the proteins (Fig. 7d and e). This type of allosteric communication between the domains, may help us understand transfer of signal associated with torque balance induced by the b subunit in response to the rotation in the γ and ε subunits and may relate to conformational changes associated with the production and release of ATP.

### 3.7 Alpha4Beta2 nicotinic receptor

**Fig. 8.**
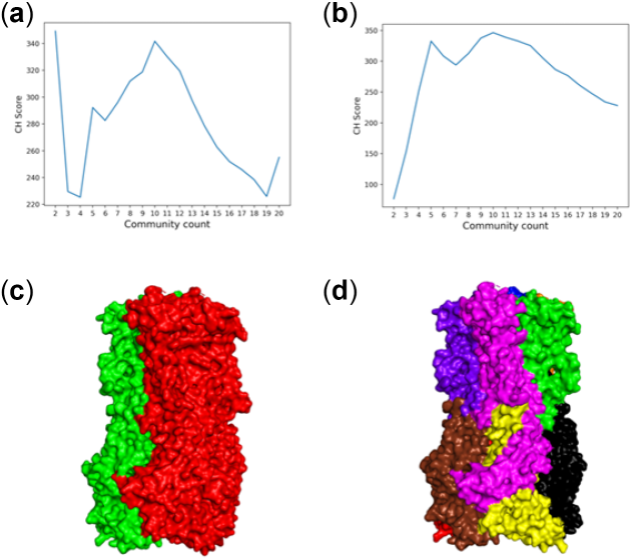
Dynamic communities in human Alpha4Beta2 nicotinic receptor, (**a**) two community in the 3α:2β assemblage (PDB ID: 6CNK), (**b**) 10 communities in the 2α:3β assemblage (PDB ID: 6CNJ). Here, each color represents a different community, (**c**) 3α:2β assemblage communities, (**d**) 2α:3β assemblage communities. The 3α:2β assemblage shows the highest peak in the CH scores for two communities and a second highest peak for 10 communities, whereas the 2α:3β assemblage shows the highest peak for 10 communities, with the CH score for two communities significantly lower, indicating that the 2α:3β assemblage has additional motions not available to the 3α:2β assembly

Human α_4_β_2_ nicotinic receptor is an acetylcholine receptor, abundantly found in human brain, which comprised α4 and β2 subunits. It forms a pentameric assembly and occurs in two different stoichiometric forms, 2α:3β and 3α:2β (Walsh *et al.*, 2018). Both assemblages are known to be functional, but they have different levels of ligand binding affinities (Morales-Perez *et al.*, 2016). Both are involved in a fast chemical communication pathway regulating the neurotransmitter-gated ion channels. Ratios of the two assemblages are commonly associated with nicotine addictions. Many studies describe its characteristics as an ion-gated channel, its pharmacology and the associated neurobiology, as well as serving as a therapeutic target for neuromuscular diseases and epilepsy (Morales-Perez *et al.*, 2016). 2α:3β shows a ∼100-fold higher affinity for acetylcholine and nicotine (Morales-Perez *et al.*, 2016). Here, we show that the 3α:2β assemblage forms two communities that has the highest CH score of 349, whereas 2α:3β is distributed into 10 communities with a CH score of 346 (Fig. 8). Alternatively, 3α:2β shows 10 communities with the second highest CH score of 341, although there is a higher CH score for two communities in 3α:2β compared with two communities for 2α:3β (Fig. 8a and b), indicating a notable change in the residue dynamic cross-correlations, therefore suggesting a significant difference in the motions for the two cases. Moreover, such a high CH score in 3α:2β for two communities as compared with 2α:3β (Fig. 8a and b), also indicates a loss of degrees of freedom in the 3α:2β assemblage, which may limit the possible conformational changes upon binding and consequently its relatively lower binding affinity. 3α:2β shows a second highest CH score peak at total community count 10, which may represent an alternate community arrangement in the structure. Similarly, 2α:3β conformational state shows nine communities with second highest CH score and five communities with third highest CH score, indicating alternate community arrangements in the protein.

## 4 Conclusions

Not only can DCI detect tightly packed and dynamically connected regions of a protein (domains), but it can also enable us to identify residues involved in hinges associated with protein open-closed transitions, find communities that directly communicate allosterically within proteins, and the conformational dynamics changes resulting from ligand binding as shown for hemoglobin. Protein functions are regulated by correlated motions among the residues. Therefore, the residue motion correlations, when combined with a data-driven clustering parameter estimation, enables DCI to detect the communities which are essential for a wide range of biological functions. Representations of protein dynamics as dynamic communities derived with DCI can help in the understanding the functionally important, strongly correlated protein motions and their functional relationships. And we have shown here that we are able to identify protein functional domains, detect cryptic pockets and aid in understanding allosteric relationships for a wide range of different types of proteins.

## Supplementary Material

btac159_Supplementary_Data
