# Supplementary material for: Coarse-graining protein structures into their dynamic communities with DCI, a dynamic community identifier
Source: Bioinformatics. 2022 Mar 17;38(10):2727–33. doi: 10.1093/bioinformatics/btac159 (PMC9113273; doi:10.1093/bioinformatics/btac159)
Supplement: btac159_Supplementary_Data [file btac159_supplementary_data.zip › supplmentary_material_DCI (1).pdf]

Supplementary Material for

Coarse-Graining Protein Structures into Their Dynamic Communities with DCI, A Dynamic Community Identifier

Ambuj Kumar, Pranav M. Khade, Karin Dorman, and Robert L. Jernigan

Robert L. Jernigan

Email: jernigan@iastate.edu

This PDF file includes:

- Table S1 to Table S2
- Figures S1 to S18
- Legends for Movies S1 to S5

Supplementary tables

Table S1: Domain residues predicted using DCI were matched with domain residue annotations obtained from SCOP database. Here DCI was able to locate the known domain regions in 73 out of 98 using optimal number of communities. Moreover, DCI was able to detect protein domains in remaining 23 examples using greater than the optimal number of communities, where the above predictions resulted in relatively larger domains as compared to the known domain length. DCI failed to detect the domains in two examples (highlighted in red), as the community boundaries were far from the actual domain residue boundaries.

**PDB**	**Protein name**	**Known domain residues (With protein chain ID)**	**Predicted domain residues (With protein chain ID)**	**Domain ID**	**Optimal number of communities**	**Protein residue length**
1a3q	Nuclear factor NF-kappa-B p100 subunit	A:227-327	A:228-327	8054870	2	285
1a3q	Nuclear factor NF-kappa-B p100 subunit	A:37-226	A:37-227	8027852	2	285
1b7f	Protein sex-lethal	A:205-289	A:206-289	8022953	2	167
1b7f	Protein sex-lethal	A:123-204	A:123-205	8022954	2	167
1bf2	Isoamylase	A:1-162	A:1-455 (A:1-160 with communities = 4)	8054932	2	750
1cgt	Cyclomaltodextrin glucanotransferase	A:495-579	A:497-556	8054908	6	684
1ciu	Cyclomaltodextrin glucanotransferase	A:496-578	A:497-683 (A:497-595 with communities=6)	8054914	3	683
1clc	Endoglucanase D (Fragment)	A:35-134	A:35-156	8054894	3	541
1cvr	Gingipain R2	A:351-432	A:354-432	8055052	4	432
1cxl	Cyclomaltodextrin glucanotransferase	A:497-583	A:489-598	8054904	6	686
1cyg	Cyclomaltodextrin glucanotransferase	A:492-574	A:492-574	8054910	7	680
1ea9	Cyclomaltodextrinase	C:1-121	C:1-135	8054922	3	583
1EDQ	Chitinase A	A:24-132	A:24-146	8054944	3	540
1EH9	Malto-oligosyltrehalose trehalohydrolase	A:1-90	A:1-107	8054934	5	557
1EIB	Chitinase A	A:24-132	A:24-142	8054946	4	540
1EX0	Coagulation factor XIII A chain	A:7-190	A:7-183	8055026	6	705

1F1S	Hyaluronate lyase	A:171-248	A:171-462 (A:171-269 with communities=5)	8054900	3	814
1G0D	Protein-glutamine gamma-glutamyltransferase 2	A:6-140	A:6-144	8055036	3	666
1GOF	Galactose oxidase	A:538-639	A:186-639 (A:532-639 with communities=5)	8054886	2	639
1GVI	Maltogenic amylase	A:1-123	A:1-130	8054920	5	588
1H6U	Internalin H	A:263-343	A:155-343 (A:238-343 with communities=3)	8055076	2	308
1IMH	Nuclear factor of activated T-cells 5	C:368-468	C:366-468	8054860	2	281
1J0H	Neopullulanase	A:1-123	A:1-146	8054928	3	588
1JMX	Quinohemoprotein amine dehydrogenase 60 kDa subunit	A:282-363	A:176-494 (A:289-364 with communities=4)	8055064	2	493
1M7X	1,4-alpha-glucan branching enzyme GlgB	B:117-226	B:117-480 (A:117-227 with communities=5)	8054942	2	587
1NFI	Transcription factor p65	A:190-314	A:194-314	8054878	2	295
1NH8	ATP phosphoribosyltransferase	A:211-284	A:212-284	8043818	2	276
1NH8	ATP phosphoribosyltransferase	A:1-210	A:1-211	8031438	2	276
1NOL	Hemocyanin II	A:380-628	A:381-628	8025670	2	607
1OGP	Sulfite oxidase	A:263-389	A:263-389	8055004	2	388
1OKE	Genome polyprotein	A:298-394	A:299-394	8054972	5	394
1OOA	Nuclear factor NF-kappa-B p105 subunit	A:251-350	A:246-350	8054868	2	313
1P7H	Nuclear factor of activated T-cells, cytoplasmic 2	L:576-678	A:575-678	8054858	2	286
1PAM	Cyclomaltodextrin glucanotransferase	A:497-582	A:497-686 (A:496-598 with communities=6)	8054912	3	686
1PBY	Quinohemoprotein amine dehydrogenase 60 kDa subunit	A:274-351	A:188-355 (A:281-355 with communities=5)	8055062	3	489
1QBA	Chitobiase	A:781-885	A:730-885	8054902	5	858
1QFH	Gelation factor	A:646-749	A:646-759	8055038	2	212
1QFH	Gelation factor	A:750-857	A:760-857	8055400	2	212
1QHO	Maltogenic alpha-amylase	A:496-576	A:494-578	8054918	6	686

1RQ5	Glucanase	A:208-305	A:208-328	8054898	5	602
1SVB	Genome polyprotein	A:303-395	A:301-395	8054966	5	395
1SVC	Nuclear factor NF-kappa-B p105 subunit	P:251-353	P:251-353	8054864	2	311
1TG8	Genome polyprotein (Fragments)	A:298-395	A:272-395	8054974	5	390
1TXK	Glucans biosynthesis protein G	A:397-511	A:254-516 (A:390-516 with communities=4)	8054950	2	480
1ULV	Glucodextranase	A:687-775	A:385-772 (A:694-772 with communities=6)	8054948	3	1019
1URZ	Polyprotein (Fragment)	A:303-401	A:287-401	8054968	4	382
1UT9	Endoglucanase (Fragment)	A:208-305	A:208-327	8054896	4	605
1W8O	Sialidase	A:403-505	A:398-647 (A:398-516 with communities=4)	8054892	2	601
1WZL	Neopullulanase 2	A:1-120	A:1-131	8054926	3	585
2A9A	Sulfite oxidase	A:344-459	A:352-466	8055002	3	371
2ALA	Structural polyprotein	A:293-380	A:294-384	8054996	6	384
2B6B	Genome polyprotein	A:298-394	A:276-394	8054982	5	389
2B20	Enterochelin esterase	A:3-150	A:3-161	8055080	2	384
2BHZ	Malto-oligosyltrehalose trehalohydrolase	A:14-110	A:14-215 (A:14-106 with communities=5)	8054938	2	581
2FHF	Pullulanase	A:163-287	A:32-296 (A:172-296 with communities=7)	8091360	3	1052
2FHF	Pullulanase	A:288-402	A:297-732 (A:297-429 with communities=7)	8091362	3	1052
2JRS	RNA-binding protein 39	A:26-108	A:23-59, A:60-108	8061922	3	108
2OMX	InlA (Fragment)	A:420-496	A:346-497 (A:419-497 with communities=5)	8055070	3	462
2RAM	Transcription factor p65	A:192-291	A:194-291	8054874	2	273
3BRD	Suppressor of hairless protein homolog	A:542-660	A:542-661	8054882	3	427
3CMG	Putative beta-galactosidase	A:609-691	A:595-698	8090626	4	661
3DO7	Transcription factor RelB	A:280-378	A:281-383	8054204	2	297
3FN9	Putative beta-galactosidase	D:602-687	D:473-687 (D:578-687 with communities=5)	8090655	2	672
3GZK	Endoglucanase	A:7-84	A:7-124 (A:7-68 with communities=8)	8101113	3	531

3HLK	Acyl-coenzyme A thioesterase 2, mitochondrial	A:58-194	A:58-185	8046445	3	400
3K2I	Peroxisomal succinyl-coenzyme A thioesterase	A:3-132	A:3-127	8046446	3	405
3LSO	Putative membrane anchored protein	A:214-307	A:213-488 (A:213-307 with communities=3)	8045893	2	439
3MLP	Transcription factor COE1	A:251-341	A:248-386 (A:248-344 with communities=5)	8052682	2	337
3N91	Uncharacterized protein	A:30-198	A:30-194	8050952	2	310
3P02	Uncharacterized protein	A:25-177	A:25-179	8045396	2	305
3POH	Endo-beta-N-acetylglucosaminidase F1	A:42-182	A:42-181	8050954	2	425
3POH	Endo-beta-N-acetylglucosaminidase F1	A:191-476	A:182-476	8090519	2	425
3SOT	Uncharacterized protein	E:31-179	E-31-173	8045394	2	288
3SOT	Uncharacterized protein	E:180-339	E-174-339	8049793	2	288
3TP2	Pre-mRNA-splicing factor CWC2	A:135-227	A:137-227	8051795	2	225
3VMN	Dextranase	A:102-210	A:102-211	8090488	5	663
3W5N	Alpha-L-rhamnosidase	A:3-112	A:3-131	8051888	5	1029
3WNO	Cycloisomaltooligosaccharide glucanotransferase	A:42-140	A:42-177	8090477	7	700
4CUC	Beta-galactosidase	A:760-863	A:648-983 (A:744-863 with communities=6)	8090615	2	847
4CUC	Beta-galactosidase	A:867-983	A:648-983 (A:867-983 with communities=6)	8090617	2	847
4DIX	Stomatal closure-related actin-binding protein 1	A:279-365	A:279-367	8046017	2	212
4DQA	DUF1735 domain-containing protein	A:32-162	A:32-162	8050955	2	340
4FE9	Outer membrane protein SusF	A:40-159	A:40-167	8046528	3	446
4FE9	Outer membrane protein SusF	A:160-273	A:168-273	8045532	3	446
4JX0	Putative exported protein	A:42-198	A:42-196	8050953	2	293
4MB4	Chitinase 60	A:348-423	A:348-428	8045516	4	528
4WGK	Neutral ceramidase	A:642-780	A:642-780	8046380	3	670
4YPJ	Beta galactosidase	A:640-732	A:642-740	8045421	6	810

4YPJ	Beta galactosidase	A:736-847	A:741-847	8090593	6	810
5AXG	Dextranase	B:28-144	B:28-144	8090493	4	584
5F7U	Lmo2446 protein	A:33-144	A:33-401 (A:33-145 with communities 8)	8090424	3	1059
5J72	Putative N-acetylmuramoyl-L-alanine amidase,autolysin cwp6	A:460-647	A:457-647	8056034	3	638
5J72	Putative N-acetylmuramoyl-L-alanine amidase,autolysin cwp6	A:138-446	A:141-456	8056030	3	638
5U0H	J30	A:1-78	A:1-87	8101107	4	543
6B6L	Glycosyl hydrolase family 2, sugar binding domain protein	A:580-686	A:565-683	8090635	5	716
6B6L	Glycosyl hydrolase family 2, sugar binding domain protein	A:690-797	A:684-797	8090637	5	716
6DHT	Xyloglucan-specific endo-beta-1,4-glucanase BoGH9A SpeciesBacteroides ovatus ATCC 8483	A:27-115	A:27-98	8101111	6	547
6MVF	Beta-galactosidase/beta-glucuronidase SpeciesFaecalibacterium prausnitzii L2-6	A:556-632	A:431-641 (A:556-641 with communities=5)	8090665	2	643

Table S2: Protein structures for Apo and holo states with cryptic pocket binding ligand.

Apo PDB ID	Apo chain ID	Holo PDB ID	Holo chain ID	Cryptic pocket binding ligand ID
2AKA	A	1YV3	A	BIT
3CHE	A	2IUZ	B	D1H
3F74	C	3BQM	C	BQM
1ALB	A	1LIC	A	HDS
3PUW	E	1FQC	A	GLO
1R1W	A	3F82	A	353
1EXM	A	1HA3	B	MAU
1MY1	C	1FTL	A	DNQ
3KQA	B	3LTH	A	UD1

Supplementary Figures

Figure S1. CH score plot for calmodulin protein (PDB ID: 1EXR).

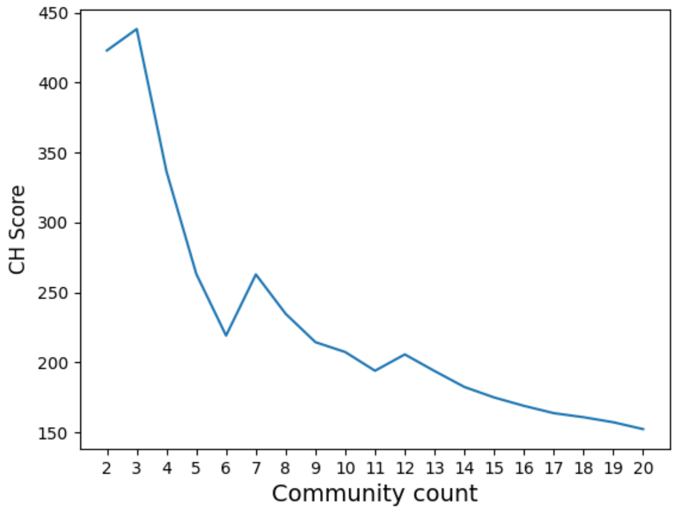

Figure S2. CH score for translocation and assembly TAMA protein (PDB ID: 4c00).

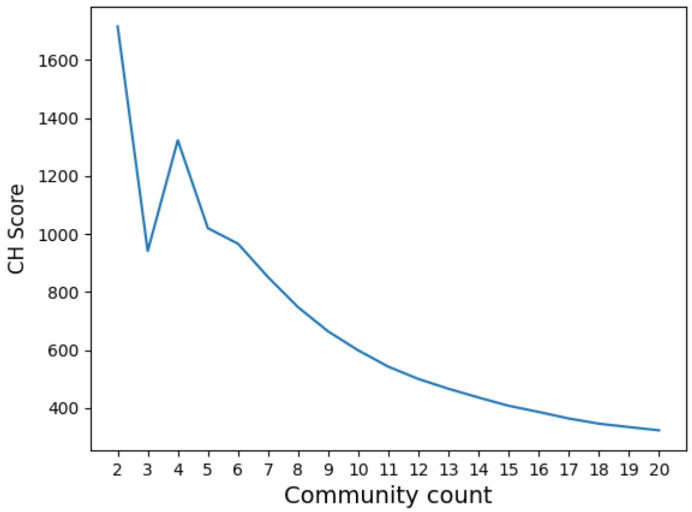

Figure S3. CH Score of glycyl-tRNA synthetase (PDB ID: 7EIV)

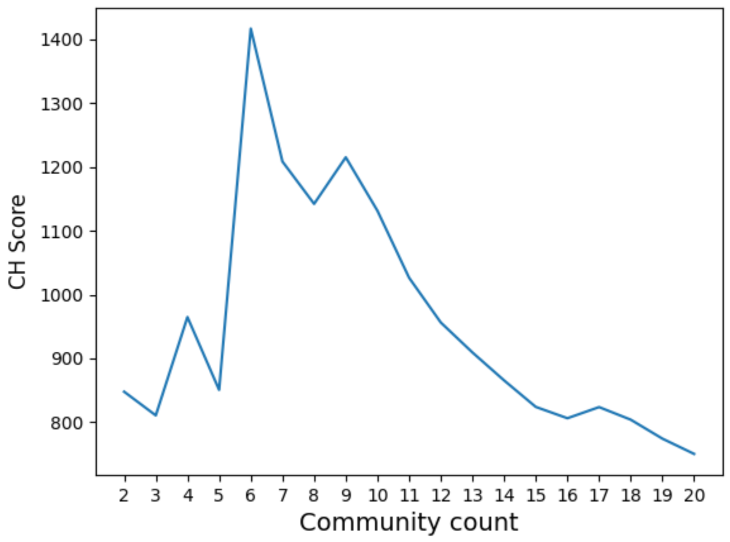

Figure S4. CH Score of autotransporter EstA (PDB ID: 3KVN)

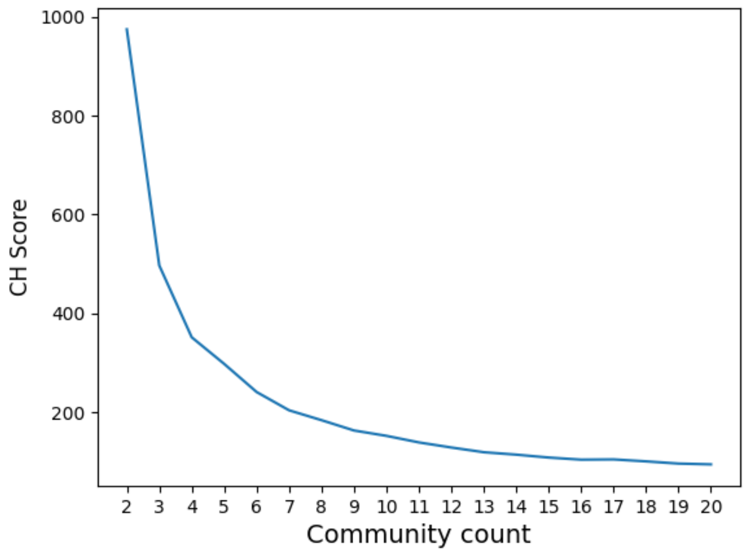

Figure S5. CH Score of autotransporter zinc transporter YiiP (PDB ID: 3H90)

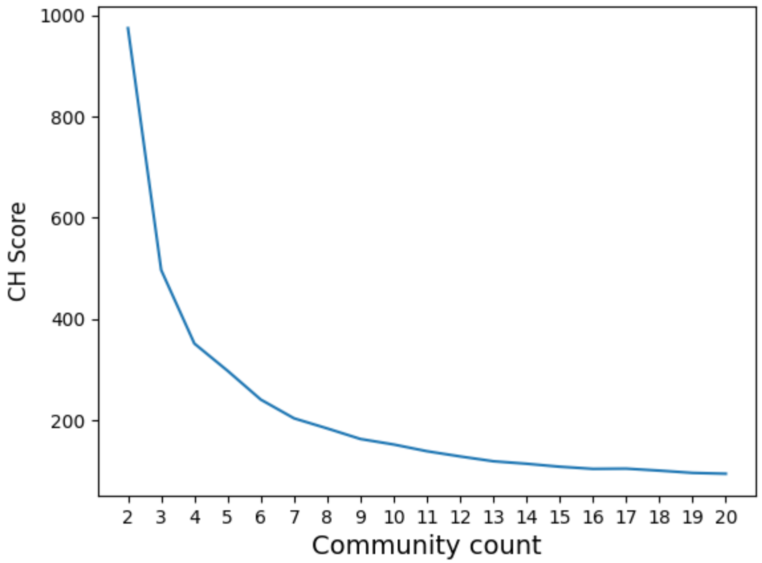

Figure S6. CH Score of autotransporter 6,7-dimethyl-8-ribityllumazine synthase (PDB ID: 2A58).

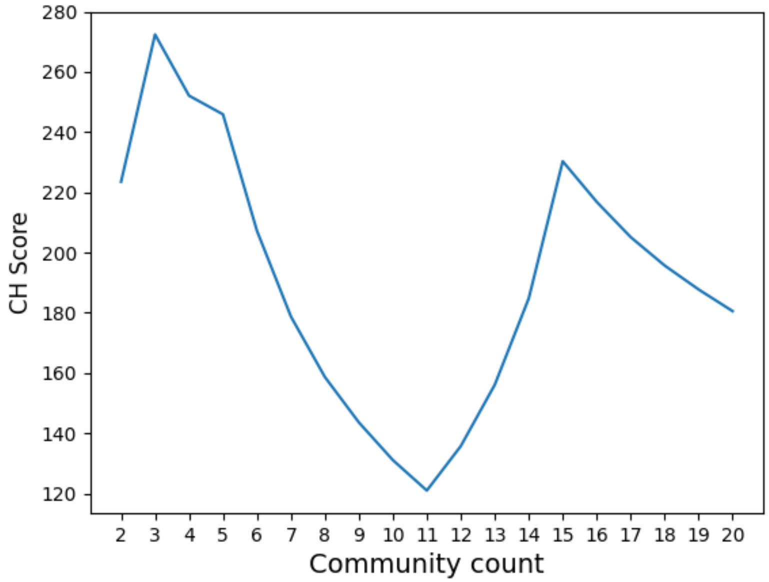

Figure S7. CH score of HLA class I histocompatibility antigen (PDB ID: 1ZSD).

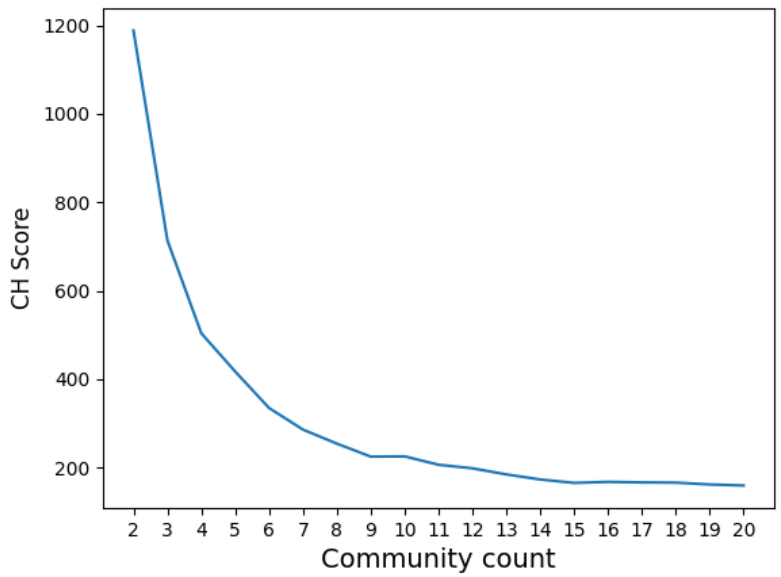

Figure S8. CH score of in Annexin protein (PDB ID: 1MCX).

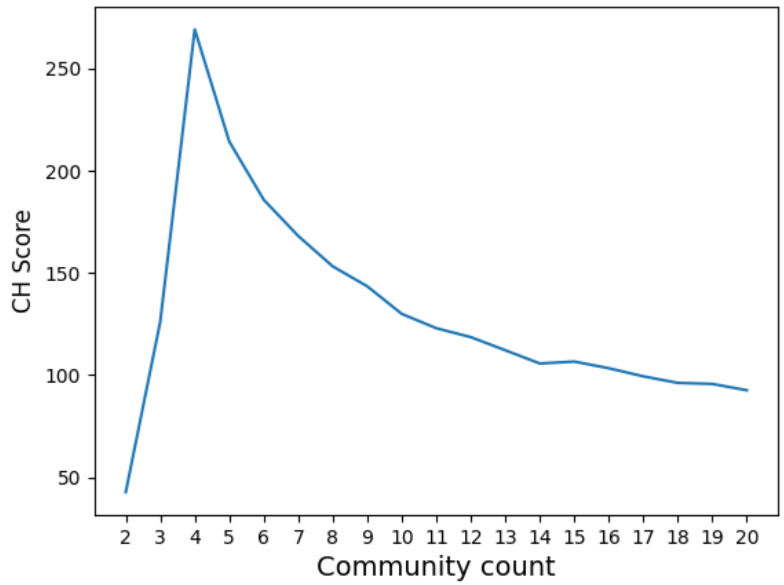

Figure S9. CH score of Inorganic pyrophosphatase (PDB ID: 1K23).

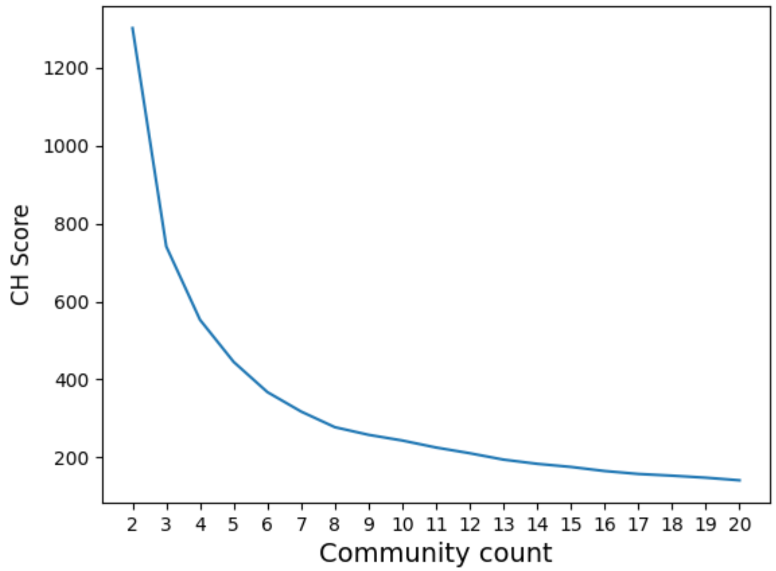

Figure S10. CH score of deoxy hemoglobin (PDB ID: 1HV4).

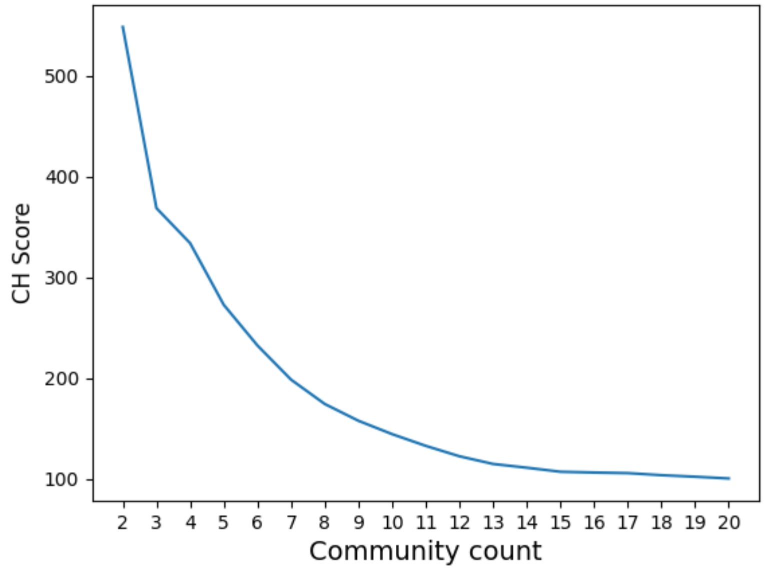

Figure S11. CH score of oxy hemoglobin (1GZX).

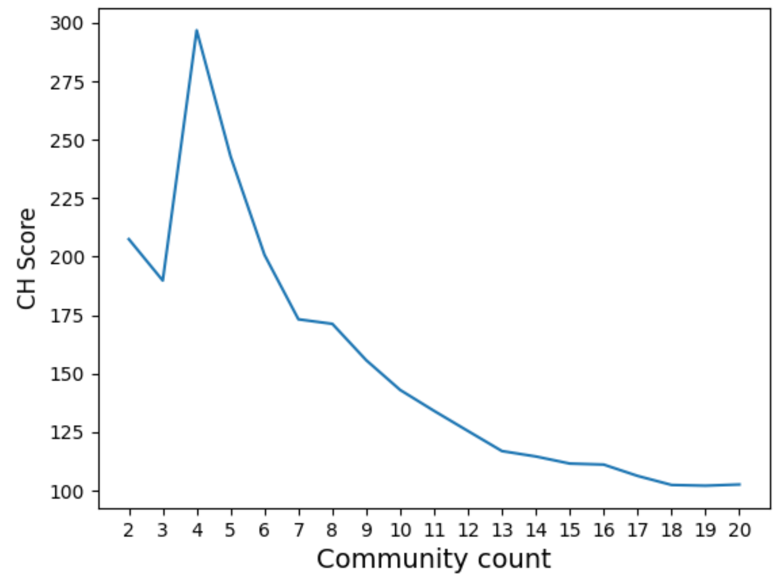

Figure S12. CH score of Glucagon class B G protein-coupled receptor (PDB ID: 5XF1).

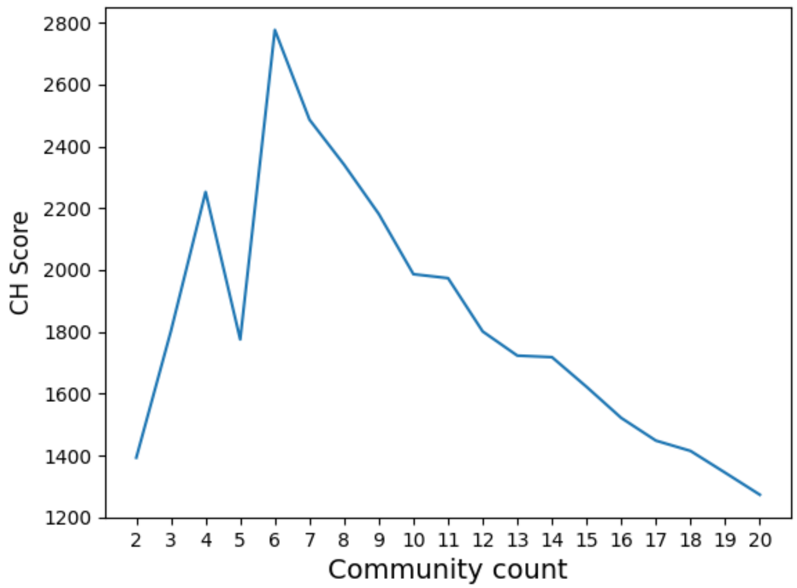

Figure S13. CH score of Metabotropic glutamate receptor 2 in complex with guanine nucleotide-binding protein complex (PDB ID: 7MTS).

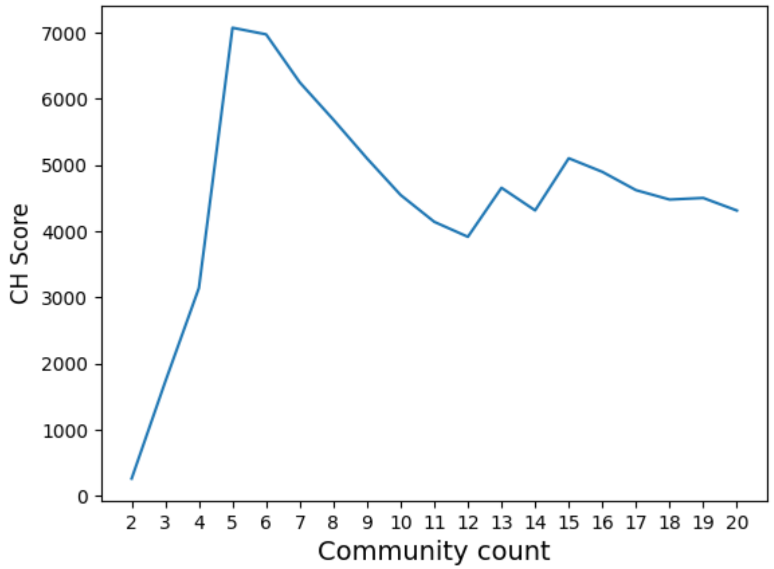

Figure S14. CH score of lipid G protein-coupled receptor (PDB ID: 3D4S).

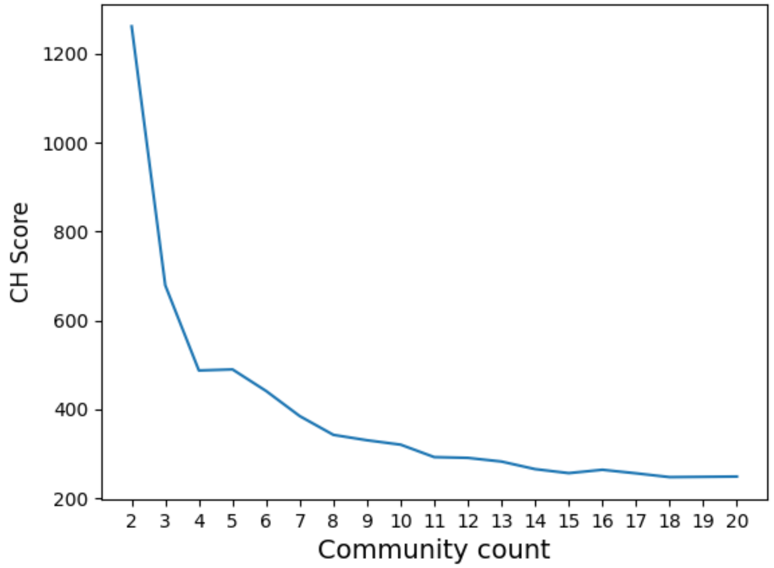

Figure S15. CH score of corticotropin-releasing factor receptor 1 protein complex (PDB ID: 6P9S).

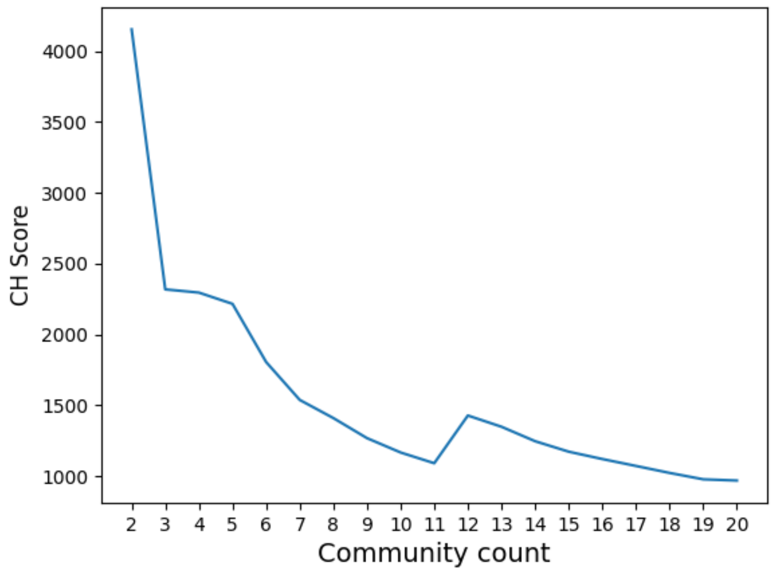

Figure S16. CH score of human cholecystokinin 1 receptor (PDB ID: 7MBY).

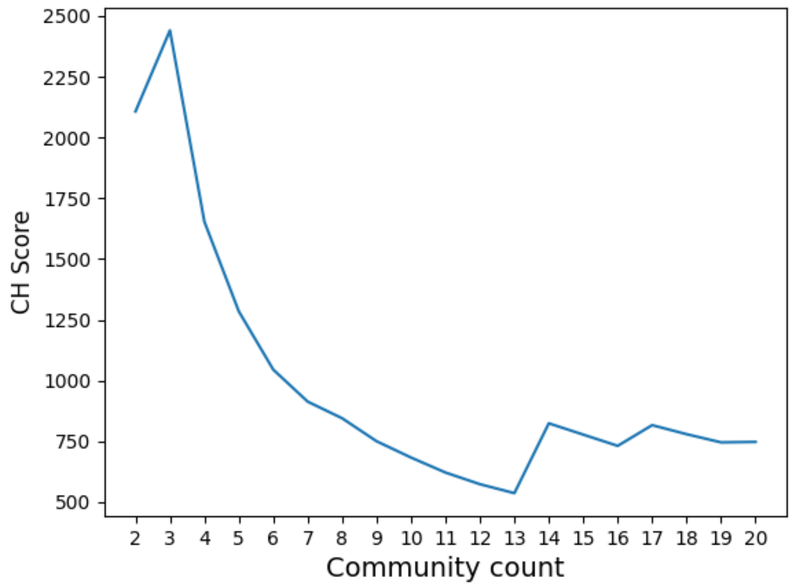

Figure S17. CH score of angiotensin II type 1 receptor bonded with TRV026 peptide and nanobody Nb.AT110i1_le (PDB ID: 6OS2).

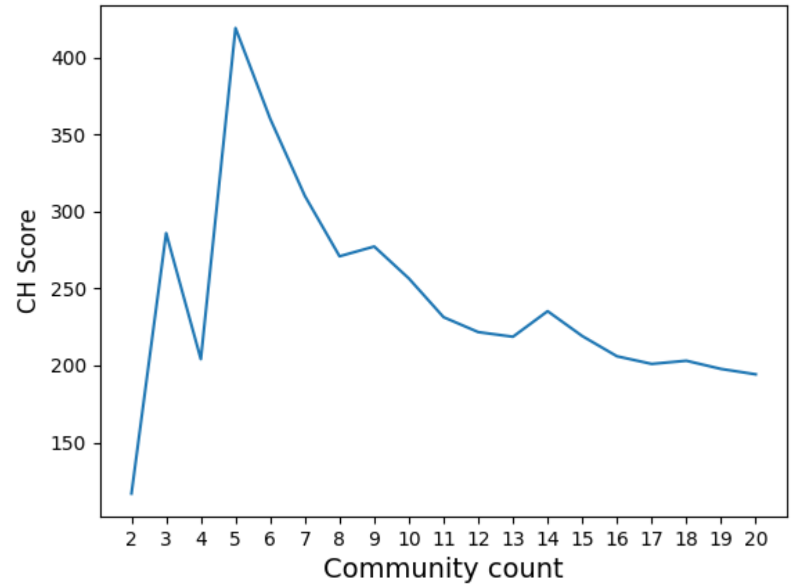

Figure S18. CH score of ATP synthase (PDB ID: 6WNR).

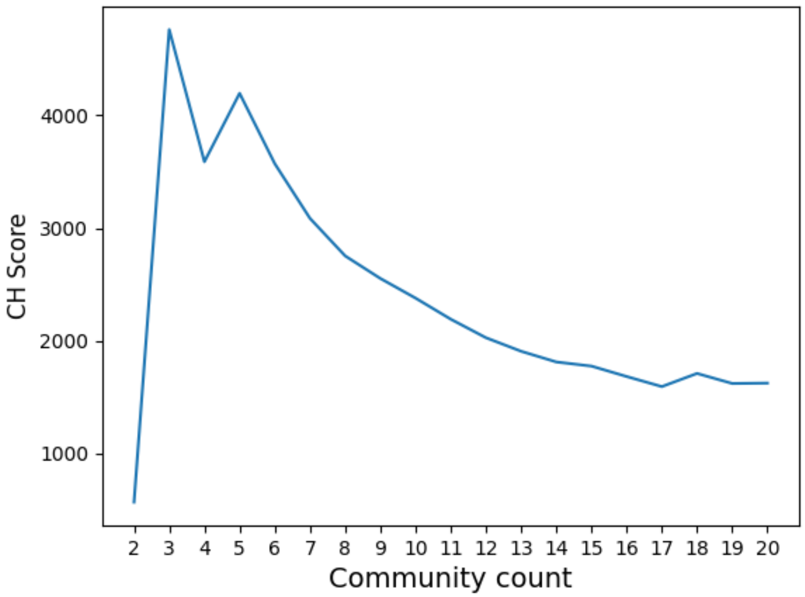

Legends for Movies

Movie S1. Domain rotation in calmodulin protein (PDB: 1EXR)

Movie S2. Hinge bending in calmodulin protein (PDB: 1EXR).

Movie S3. Open-closed transition in HLA class I histocompatibility antigen protein (PDB ID: 1ZSD).

Movie S4. Open-closed transition in Annexin protein (PDB ID: 1MCX).

Movie S5. Open-closed transition in Inorganic phosphatase protein (PDB ID: 1K23).
